# *Mycoplasma bovis* mastitis in dairy cattle

**DOI:** 10.3389/fvets.2024.1322267

**Published:** 2024-03-06

**Authors:** Aga E. Gelgie, Sarah E. Desai, Benti D. Gelalcha, Oudessa Kerro Dego

**Affiliations:** ^1^Department of Animal Science, The University of Tennessee, Knoxville, TN, United States; ^2^College of Veterinary Medicine, The University of Tennessee, Knoxville, TN, United States

**Keywords:** *Mycoplasma bovis*, *Mycoplasma bovis* mastitis, dairy cows, subclinical mastitis, virulence factors, pathogenesis, clinical mastitis, intramammary infection

## Abstract

*Mycoplasma bovis* has recently been identified increasingly in dairy cows causing huge economic losses to the dairy industry. *M. bovis* is a causative agent for mastitis, pneumonia, endometritis, endocarditis, arthritis, otitis media, and many other clinical symptoms in cattle. However, some infected cows are asymptomatic or may not shed the pathogen for weeks to years. This characteristic of *M. bovis*, along with the lack of adequate testing and identification methods in many parts of the world until recently, has allowed the *M. bovis* to be largely undetected despite its increased prevalence in dairy farms. Due to growing levels of antimicrobial resistance among wild-type *M. bovis* isolates and lack of cell walls in mycoplasmas that enable them to be intrinsically resistant to beta-lactam antibiotics that are widely used in dairy farms, there is no effective treatment for *M. bovis* mastitis. Similarly, there is no commercially available effective vaccine for *M. bovis* mastitis. The major constraint to developing effective intervention tools is limited knowledge of the virulence factors and mechanisms of the pathogenesis of *M. bovis* mastitis. There is lack of quick and reliable diagnostic methods with high specificity and sensitivity for *M. bovis*. This review is a summary of the current state of knowledge of the virulence factors, pathogenesis, clinical manifestations, diagnosis, and control of *M. bovis* mastitis in dairy cows.

## 1 Introduction

*Mycoplasma bovis*, which was formerly known as *Mycoplasma agalactiae* subsp. *bovis* (1), is a causative agent of several diseases in cattle and other farmed ruminants including mastitis, pneumonia, endocarditis, arthritis, otitis, meningitis, and reproductive problems both in bulls and cows (2–6). *M. bovis* mastitis is an emerging dairy cattle disease that poses a significant challenge globally due to its highly contagious nature and resistance to antimicrobials (7). Despite the isolation of various *Mycoplasma* spp. from milk samples of cows with mastitis, *M. bovis* is the most common causative agent (8, 9).

In the United States of America (U.S.) alone, economic losses due to *M. bovis* mastitis is estimated to be above $100 million annually (10). This mainly results from increased somatic cell counts (SCC), decreased milk production, culling, and treatment costs. Since SCC is an indicator of milk quality and udder health, SCC beyond legal limits in the U.S. results in regulatory measures such as suspension of operation permits (11). Culling of infected cows has proven costly in the U.S. with the rate reaching as high as 70% (12). The prevalence of *M. bovis* mastitis is high in large dairy operations especially in the U.S. dairy industry where several large scale dairy operations exist (13, 14). Currently adopted mastitis control programs which involve environmental sanitation, proper milking procedures and udder health (15) are partly ineffective due to transmission of *M. bovis* through respiratory route and semen or seminal fluid (16, 17) as well as calf to cow or *vice-versa* (18). Given the difficulty in diagnosis due to the fastidious growth nature and subclinical infection, with infected cows apparently appearing healthy, the disease can remain undetected for long time in dairy farms with significant economic losses (19).

Although *M. bovis* mastitis was first reported in the early 1960s (2) and has since been problematic, effective control tools such as vaccine or prophylactic therapy or treatment is yet to be developed. This is largely attributed to the knowledge gap in the critically important virulence factors and pathogenesis mechanisms of *M. bovis* mastitis.

With increasing antimicrobial resistance problem (20), accurate diagnosis is crucial to avoid the use of broad-spectrum antimicrobial drugs and to conduct targeted treatment. Among the challenges in the control and prevention of *M. bovis* infections is developing effective and sustainable control tools and rapid and reliable pen-side diagnostic tool with high specificity and sensitivity that can be used at farm level (21). This review is a succinct summary of current state of knowledge in virulence factors, pathogenesis, clinical manifestations, diagnosis, and control measures of *M. bovis* mastitis in dairy cows.

## 2 Virulence factors

### 2.1 Adhesion and invasion

Mycoplasmas lack cell walls and have exposed membrane proteins. The exposed membrane proteins primarily interact with host surfaces and enable the bacteria to adhere to the host mucosal surfaces and are also necessary for the bacteria to acquire nutrients from their surroundings and evade their host’s immune response (22).

Adhesion is an essential virulence attribute in mycoplasmas since adhesion mutants are avirulent (23). *M. bovis* utilizes a 48.8 kDa receptor TrmFO that binds to host fibronectin, an extracellular matrix glycoprotein (24). Other isolates also express key adhesins such as α-enolase, a hypothetical lipoprotein with adhesin activity (P27), variable surface lipoprotein A (VpmaX), and fructose-1,6-biophasphate aldolase (25, 26). A cytoadhesive surface exposed protein in certain strains is expressed to surmount the highly tight epithelial junctions during infection in organs such as lung (27). Attachment only, however, does not constitute internalization as some *M. bovis* cells were shown adhered onto and others internalized into calf turbinate cells in a single *in vitro* infection (28, 29). Burki and co-authors showed that *M. bovis* enters host cells through non-classical endocytic pathway (28) which involves invagination of plasma membrane to internalize pathogens (30). This pathway is used by host cells to uptake various fluids and solutes but *M. bovis* takes this to its advantage. Autophagy is a highly conserved self-destructive process in eukaryotic cells aimed to remove faulty organelles, misfolded proteins and pathogens (31). However, *M. bovis* prevents autophagy in bovine mammary epithelial cells to replicate in an intracellular environment while also avoiding clearance by host immune responses and antimicrobial agents (32, 33). *M. bovis* exerts this effect through blocking autophagic flux which involves recognition of intracellular *M. bovis* by receptors, delivery to enzyme-bound membranes and final transport to lysosomes, a degradation machinery (33).

### 2.2 Variable surface proteins

Among the characteristics that increases the virulence of *M. bovis* is the collection of immunodominant variable surface proteins (Vsps). These surface lipoproteins are highly variable in their size and coding sequences (34, 35). Due to different surface lipoprotein variants potentially interacting with the host immune system at any given time, immune responses against these Vsps are not effective. Furthermore, the pathogenicity of *M. bovis* is significantly increased because these Vsps enable it to avoid detection and clearance by a host immune system (36).

#### 2.2.1 Nucleases

Mycoplasmas do not have biosynthetic mechanisms to synthesize nucleic acid precursors and depend on cellular nucleases to generate nucleotide precursors (37). Nucleases are components of the mycoplasmal membrane that hydrolytically cleave the phosphodiester backbone of DNA and they play important role in acquisition of the host nucleic acids. Various *Mycoplasma* cellular nucleases have been characterized which are believed to be important for generating nucleotides and hence expected to contribute to virulence (38). Endonucleases cleave the phosphodiester bond in the middle of chains within the polynucleotide whereas the exonucleases selectively cleave the polynucleotide chain either at the 5′ or 3′ ends (39).

#### 2.2.2 Biofilm formation

It has been demonstrated that *M. bovis* produces biofilms; however, the level of adhesion and effectiveness of the biofilms vary between different strains depending on the surface lipoprotein (Vsp) expression. Biofilms of *M. bovis* increase heat resistance at 50°C and desiccation tolerance but are not any more resistant to antimicrobials compared to planktonic cells (40).

#### 2.2.3 Nucleomodulin secretion

Nucleomodulins are effector proteins secreted by bacteria that can interact with the host DNA and serve to regulate gene transcription to favor the pathogenesis of the bacteria (41). The MbovP475 lipoprotein is secreted by *M. bovis* and binds the promoters of the cell cycle central regulatory genes, CRYAB and MCF2L2 genes and downregulates their expression in bovine macrophage cell line (42) resulting in decreases in bovine macrophage cell line viability.

### 2.3 Metabolites

*M. bovis* synthesizes hydrogen peroxide (H_2_O_2_) which has the potential to react with iron and copper ions to produce cytotoxic hydroxyl radicals (43). This is possible through the NADH oxidase enzyme expressed by *M. bovis* which reduce oxygen to H_2_O_2_ on top of its adhesin role (44). Reactive oxygen species produced by *M. bovis* can cause varying degree of damage in bovine mammary epithelial cells including apoptosis (45).

## 3 Pathogenesis of *Mycoplasma bovis* mastitis and clinical symptoms

The most common clinical manifestations of *M. bovis* mastitis includes udder swelling and abnormal milk appearance ranging from watery and flaky milk to thick purulent inflammatory fluid (46). However, some cows may show no outward symptoms although having subclinical mastitis with or without shedding the bacterium (47–50). *M. bovis* mastitis causes an increase in SCC. Among the initial reactions during intramammary challenge infection include high SCC and acute phase proteins (51). These authors reported increased production of serum amyloid A and lipopolysaccharide -binding protein in experimentally induced *M. bovis* intramammary infection. It is possible that *M. bovis* spreads from one site of infection to another via blood circulation. This was demonstrated by isolation of *M. bovis* from previously uninfected cows which were challenged experimentally by the intramammary route (46). Although shedding and re-infection is a possibility due to contagious nature of the organism, *M. bovis* from a milk of mastitic cows had identical pulsed field gel electrophoresis pattern with those isolated from other body parts such as eyes, nasal cavities and ears which is strong indication of internal dissemination (52).

Transmission of *M. bovis* has been linked to colostrum, milk, semen, air-borne, and intrauterine routes (16, 53–55). Udder-to-udder is thought to be the primary route by which the infection is transmitted between cows (12, 56). Although *M. bovis* intramammary infection is widespread in lactating dairy cows, it is also not uncommon in dry cows (57).

Several aspects of *M. bovis* mastitis differs from other major mastitis pathogens. Unlike coliform mastitis, which is environmental, *M. bovis* mastitis is contagious which means it can be transmitted from infected cows to healthy cows during milking time. The major contagious mastitis pathogens include *Staphylococcus aureus, Streptococcus agalactiae, and Mycoplasma* spp. (13). Compared to *Staphylococcus aureus* and *E. coli*, *M. bovis* weakly affects mRNA expression in bovine mammary epithelial cells (58). According to the USDA, *M. bovis* is more prevalent in large (500+) dairy operations compared to other contagious mastitis pathogens such as *Streptococcus agalactiae* (13).

## 4 Host immune responses against *Mycoplasma bovis* infection

### 4.1 Innate immunity

Epithelial cells are the major cell types in the bovine mammary gland and are the first line of defense. Upon first encounter, *M. bovis* adhere to and invade mammary gland epithelial cells (59) and bovine mammary epithelial cells respond by upregulated expression of proinflammatory cytokines such as interleukin (IL)-6 and IL-8 and TNF-α (58, 60). Autophagy is one of the mechanisms these cells employ to eliminate invading bacteria, however under-expression of autophagy related proteins has been demonstrated in *M. bovis* infected bovine mammary epithelial cells (32, 33).

*M. bovis* mastitis is characterized by massive recruitment of neutrophils into the milk spaces of the mammary gland (61). Similarly, *M. bovis* has been shown to stimulate production of neutrophils extracellular traps (NETs) and possess the membrane nuclease, MnuA which degrades the NETs through either exo- or endonuclease activity (38, 62). In addition, another membrane nuclease of *M. bovis* (MBOV_RS02825) has been demonstrated to degrade NETs and cause apoptosis in macrophages (63). NETs are considered part of the innate immune response which have DNA as a major structural component to trap and stop pathogenic bacteria from spreading (64). Degradation of the NETs by mycoplasmal nucleases have a bifold advantage including avoiding the entrapment and opsonophagocytic killing by neutrophils and scavenging the nucleotide precursors (65). Furthermore, *M. bovis* promote neutrophil apoptosis to ensure its persistence and systemic dissemination.

*M. bovis* has been observed to elicit proinflammatory cytokine and chemokine responses in infected hosts which may weaken the host and increase the pathogenicity of the bacteria (66). Opsonization is necessary for phagocytosis of *M. bovis* by macrophages and neutrophils, but *M. bovis* can combat this through surface antigen variation, and biofilm formation (40). Macrophages kill *M. bovis* via phagocytosis up on opsonization support from IgG1 and IgG2 (67). In a study conducted to determine *M. bovis-*bovine viral diarrhea virus (BVDV) synergism during infection, *M. bovis* induced apoptosis and cytotoxicity in bovine macrophages (68). In contrary, another study reported significant reduction in apoptosis in macrophages induced by *M. bovis* possibly as a mechanism of survival (69).

*M. bovis* inhibits proliferation of peripheral blood mononuclear cells (PBMCs) to evade the immune system and cause chronic infections (70). However, in contrary another study reported an increase in the expression of TNF-α, IL-12 and IFN-γ and increased proliferative responses in PBMCs stimulated with *M. bovis* (71). Following intramammary inoculation with *M. bovis*, milk from infected quarters exhibited increased SCCs, yet there was not a significant difference between levels of PBMCs or mononuclear cells in the stimulated and unstimulated mammary lymph nodes (72). In fact, there was a decrease in the mRNA levels of innate-immunity related genes from blood mononuclear cells following intramammary infection with *M. bovis*, such as complement factor D (CFD), ficolin 1 (FCN1), and tumor necrosis factor superfamily member 13 (TNFSF13) (72). This alteration of the host transcriptome likely contributes to the chronic nature of many *M. bovis* infections.

### 4.2 Adaptive immunity

*M. bovis* antigens activate host CD4^+^, CD8^+^, γδ T- cells, B- cells, and leukocytosis (73, 74). In addition, *M. bovis* also induces IgG1and IgG2 responses (75). IgG1, however, has a lower opsonin effect which does not activate a strong humoral immune response in the host and resulting in persistence of *M. bovis* infections for long periods of time (76). The effect of cytokines, such as interferon gamma (IFN-γ), that encourage cell death but also cytokines that characterize the Th2 response and slow recovery of tissues likely contribute to the pathogenicity of *M. bovis* and make it more difficult for the host to recover from infections (77).

## 5 Diagnosis of *Mycoplasma bovis*

### 5.1 Culture

Microbial culture has traditionally been used to definitively diagnose *M. bovis*. However, the longer time it takes to grow makes culture unfavorable to make a rapid diagnosis. Most common specimens to diagnose *M. bovis* includes milk, bronchioalveolar lavage, deep nasopharyngeal swabs, joint fluids, and semen. *Mycoplasma* plates are incubated at 37°C in 5% CO_2_ for 7–10 days and colonies are typically characterized by a ‘fried egg appearance’ when observed under light microscope (78). This is unfavorable where rapid diagnosis is needed to isolate infected animals, limit further dissemination of infections and commence appropriate antibiotic therapy (79). Due to the longer days required by *M. bovis*, sometimes the colonies are overgrown by other bacteria to the extent that they cover *M. bovis* colonies and makes it difficult to observe under microscope (80). Not only they are overgrown on the plates, but they are also overgrown in the milk since mycoplasmas have limited capability to multiply in milk (81).

Mycoplasmas have one of the smallest genome sizes (0.58–1.38 Mbp) which likely renders them needy of nutrients such as amino acids and fatty acids (22, 82). Another challenge with *M. bovis* culture is its detection limit which is greater than or equal to 272 CFU/mL (83) meaning any number of mycoplasma cells less than the detection limit could possibly be overlooked. Culture also fails to differentiate other non-pathogenic mollicutes such as *Acholeplasma* which exhibit same ‘fried egg’ appearance as *M. bovis* and can potentially contaminate *M. bovis* samples (84).

Bulk tank milk samples were used in several studies to estimate prevalence and other research purposes (85–88). The major problem with bulk tank milk sampling is collecting a representative amount of sample out of 200 to 2,600 gallons of tanks which could massively dilute the amount of *Mycoplasma* cells. Usually, about 10–40 mL of milk sample is collected (85, 87), centrifuged and only 100–200 μL are spread on the *Mycoplasma* plates. Some laboratories culture milk samples directly (12), however it has been demonstrated that centrifugation and resuspension can potentially increase the rate of recovery (89). Occasionally, enrichment of milk samples in *Mycoplasma* broth before culture is practiced (90). Milk samples need to be processed immediately after collection to maximize the likelihood of positive diagnosis or need to be frozen although freezing has been shown to cause 1–2 log_10_ reduction (91). In addition, multiple sampling is greatly advised due to intermittent shedding behavior of the pathogen in mastitis cases (49, 92). However, there are also studies which were conducted based on one-time bulk tank milk sampling (85, 93).

### 5.2 Polymerase chain reaction

PCR is such a sensitive method in *M. bovis* diagnosis that it detects as low as 10 CFUs from broth cultures (94, 95). Compared to culture, enzyme-linked immunosorbent assay (ELISA), sodium dodecyl sulfate-polyacrylamide gel electrophoresis (SDS-PAGE), and nucleic acid hybridization, PCR was found to be superior in terms of high specificity, sensitivity and rapidity (96). However, expensive reagents and equipment are the major setbacks to apply in small-scale laboratories and farm environments. The application of PCR in *M. bovis* diagnosis brought about several advantages. It bypasses the tiresome culturing and samples can directly be screened for *M. bovis* DNA. In comparison to the culture method which only detects live organisms, PCR can detect the DNA from dead microorganisms which can have several implications. This is true, for instance, perhaps if milk samples are stored in a freezer for long time and *M. bovis* cells are no longer viable (91). In some cases, pre-enrichment is recommended before performing PCR assays for as many as 4 days (97). This might save time and resource in terms of avoiding culturing negative samples, however is not helpful when rapid diagnosis is needed.

*UvrC* gene has widely been used as a target in conventional and real-time PCR in *M. bovis* detection in bulk tank milk and lung samples (87, 98, 99). It is one of housekeeping genes in *M. bovis* which encodes an enzyme that mediates excision DNA repair system (82, 100). Furthermore, this gene has been recommended for use in routine laboratory diagnosis of *M. bovis* (101). Realtime PCR has been shown to be more sensitive than conventional PCR and was able to amplify as low as 40 copies of the target gene (98). The CT values in real-time and quantitative PCR (qPCR) can be used as a predictive tool for *M. bovis* isolation (97).

A multiplex PCR which separately detects different species such as *M. bovis, M. bovigenitalium,* and *M. californicum* in a same sample which would greatly reduce efforts and time spent to perform multiple reactions (102). Furthermore, bacterial species such as *Mannheimia hemolytica* are usually isolated alongside *M. bovis* in respiratory diseases (103). Multiplex quantitative PCR has been proven to specifically detect and quantify these respiratory pathogens (104, 105). Nested-PCR, where two sets of primers are employed, is preferred to increase sensitivity and specificity of *Mycoplasma* detection (106, 107). PCR has also been employed to identify antibiotic resistance genes in *M. bovis* (108, 109).

### 5.3 Indirect and direct ELISA

Unlike culture and PCR, ELISA detects the anti-*M. bovis* antibodies in the host serum or milk from past or recent infections as a result of humoral immune responses. ELISA has widely been used on bulk tank milk samples to detect anti-*M. bovis* antibodies in the milk for diagnostic, prevalence, and retrospective studies (85, 88, 110, 111). A MilA ELISA with a specificity and sensitivity as high as 94.2 and 96.6%, respectively, has been used to estimate *M. bovis* mastitis prevalence from bulk tank milk samples (112). This ELISA, in which MilA membrane protein was used as a coating antigen, was first developed in Australia (113). Furthermore, the *milA* gene was expressed on the surface of a phage and was used as antigen in indirect ELISA which was reported to be inexpensive and convenient compared to the MilA peptide protein (114). However, bulk tank milk is not a fully representative sample for a herd since *M. bovis* can cause various disease manifestations in various age groups (115). This necessitates considering blood samples in certain cases where the young stock is suspected to harbor the infection or they are being newly introduced to herd. ELISA might not necessarily indicate active *M. bovis* infection as positive ELISA results turns negative in PCR when both methods were used in same herds (85, 116). Thus, ELISA can be used as a biosecurity tool before introducing newcomers to the herd (110) and as a surveillance tool to monitor and confirm eradication of *M. bovis* infections in some nations (117). It also plays role in testing immunogenicity of novel proteins in a study conducted to investigate *M. bovis* pathogenesis and its protective antigens (118).

The use of Indirect ELISA to detect anti-*M. bovis* antibodies is challenged by cross-reactivity from other *Mycoplasma* spp. such as *M. agalactiae* (119). Indirect ELISA might not be as rapid as desired sometimes, since it takes 1–2 weeks for the animal to mount humoral immune response (seroconversion) and results could possibly turn negative in this time window (78), thus it needs to be used in conjunction with other tests. Once antibodies are produced by the host, however, ELSIA is not affected by the intermittent shedding behavior of *M. bovis* in the milk which indicates it is important to use culture, PCR and/or ELISA together, whenever possible, since they complement each other. Unlike indirect ELISA, the use of direct ELISA in *M. bovis* diagnosis is limited. There are not many direct ELISAs reported, however one study reported membrane protein P48 based monoclonal antibodies has been shown to specifically detect *M. bovis* without cross-reactivity with related species such as *M. agalactiae* (120).

### 5.4 MALDI-TOF MS

Matrix-Assisted Laser Desorption Ionization – Time of Flight Mass Spectrometry (MALDI-TOF MS) has lately been adapted as a rapid tool to accurately diagnose microbes. The principle behind the method is formation of ions from intact bacterial cells using laser light ionization (121). Samples are obtained from colonies on agar plates, mixed with matrix solution, and introduced to a mass spectrometer ion source. Results are analyzed against archived references or with colonies from known bacteria. Following a comparison with the 16S rDNA PCR as gold standard, MALDI-TOF has been in agreement for 97.8% species-level and 99.6% genus-level for aerobes and 95.3% species-level and 100% genus-level for anaerobes which shows that it is a preferable method to diagnose bacteria of veterinary interest (122). The suitability of MALDI-TOF to distinguish between phylogenetically closest sub-species in human and ruminant mycoplasmas has been confirmed (123). Although some studies recommend MALDI-TOF as promising test for routine diagnosis of *M. bovis,* the disadvantage lies in its dependence on enrichment which can take 2 to 4 days (124, 125). The starting material for MALDI-TOF could also be a secretome extraction or a protein from *M. bovis.* Zubair et al. used extracted secretome from *M. bovis* HB0801 strain to predict 8 proteins related to a virulence which signifies the importance of MALDI-TOF in identifying potential diagnostic and vaccine targets (126).

Limited library of mycoplasmas in the MALDI-TOF databases has been reported as one of the challenges in using this method to detect *M. bovis* (125). Initial MALDI-TOF installation and instrumentation is also believed to be expensive which makes its application at large scale level difficult (127). Therefore, MALDI-TOF is commonly used more as a confirmatory technique rather than a routine diagnostic tool.

### 5.5 Loop-mediated isothermal amplification

The LAMP is a high sensitivity method which utilizes a set of 2 to 3 primers that can produce several copies (108) of target DNA in less than an hour since it bypasses the denaturation step (128). As the name indicates, LAMP amplification occurs within a constant temperature. Unlike the conventional PCR, LAMP does not need thermocyclers and can easily be done in heating block (129). Following amplification, results can be read in 2% agarose gel electrophoresis; using SYBR green I staining or based on turbidity of the reaction mixture (129, 130). *UvrC-*based LAMP in *M. bovis* has been demonstrated to have 10-fold higher sensitivity compared to PCR with 100 and 74% sensitivity and specificity, respectively, (129), however later on other researchers improved the specificity to 90.9% (130). Other *M. bovis* genes such as *oppD* (encodes oligopeptide permease D), *gltX* (glutamate transfer RNA ligase), *gyrB* (gyrase B subunit) and 16 s rRNA were also employed to show sensitive and specific detection of *M. bovis* using LAMP (131, 132).

## 6 Control and prevention measures

Globally, DNA amplification techniques used for detection and identification of bacteria have only become widely globally accessible within the last 30 years, making it difficult to trace the exact time and route by which *M. bovis* first spread around the world (94). The first definitive identification of *M. bovis* infection was in 1961 in the U.S. (2). From there, the pathogen was thought to have been spread to other countries through movement of cattle and cattle products (7).

The strategy toward the control and prevention of *M. bovis* mastitis, or *M. bovis* infection in general, depends on the country. New Zealand, which is the latest country to report *M. bovis* mastitis in 2017, prefers a nationwide complete eradication program (117). However, other endemic countries endeavor to contain infections at the farm level through culling or isolating infected animals (133). Finland, for example, pursues a voluntary control program involving farmers since *M. bovis* is regarded as one of less serious diseases (134).

Currently, the best-known method for controlling *M. bovis* is mere prevention of exposure to the pathogen and other infected cows. Screening of original herd before purchasing new cows is worthwhile as well as quarantine of new cows which adds extra layer of security. In addition, isolation and culling of infected cows are necessary measures to effectively control the disease and minimize outbreaks (42). However, advances in rapid and accessible tests to detect *M. bovis* on dairy farms are necessary to control and prevent outbreaks from occurring more effectively.

### 6.1 Use of antimicrobials

There are currently no known effective treatments against *M. bovis* available for use. One of the main reasons for this is the growing incidence of antimicrobial resistant bacterial strains. Drugs such as tiamulin, enrofloxacin, danofloxacin, and florfenicol has been reported to have low minimum inhibitory concentration against *M. bovis* (20). However, in the last 2 decades, *M. bovis* have shown less susceptibility to antimicrobial agents like fluoroquinolones (135). Furthermore, lack of cell wall makes the organism resistant to commonly used antimicrobials such as penicillin and cephalosporins. Macrolides such as tylosin and tilmicosin which were traditionally used to treat *Mycoplasma* infections has gradually become less effective (136). Recent trends indicate that antimicrobial resistance against other common antimicrobials, such as tetracyclines, has been increasing as broadly reviewed elsewhere (20). Some natural compounds have been shown to be promising and may further be developed to produce effective therapeutic options (137).

### 6.2 Vaccines

Many potential vaccine candidates have been developed but are not available for widespread commercial use. For example, autogenous vaccines have been developed (138); however, such vaccines are useful only for a single farm, limiting their potential for large-scale use. Other vaccines showed efficacy in studies but failed to elicit any protective effects in field trials as they failed to reduce the incidence of *M. bovis* cases (139, 140). There are currently no commercially available effective vaccines that prevent the incidence of *M. bovis* infection. Given the fact that *M. bovis* causes pneumonia in the feedlot cattle (141), considerable number of vaccine works has been done using feedlot cattle as a model. Prysliak and co-authors developed a sub-unit vaccine using the highly conserved glyceraldehyde-3-phosphate dehydrogenase (GAPDH) protein; however, subsequent controlled experimental efficacy evaluation showed that it did not confer protection against experimental challenge (142). Similarly, intranasal inoculation of total protein extract and membrane fractions from *M. bovis* triggered strong humoral immune responses but failed to protect against experimental challenge infection (143). Another challenge in vaccine development against *M. bovis* mastitis is strain variations of *M. bovis* (6). *M. bovis* also expresses antigenically variable surface proteins (34, 144) and thus necessitates developing vaccines from highly conserved immunogenic proteins. Despite several trials in the past, conserved immunogenic molecules which can elicit protective immune responses against *M. bovis* mastitis are yet to be identified.

## 7 Perspectives and future directions

In summary, effective control tools such as vaccine or prophylactic or therapeutic drugs against *M. bovis* mastitis are not available. Currently adopted mastitis control programs which involve environmental sanitation, proper milking procedures, and udder health are partly ineffective due to transmission of *M. bovis* through the respiratory route, semen or seminal fluid as well as calf to cow or *vice-versa*.

Rapid diagnosis is extremely important for the identification and culling of infected animals before infection spreads through the herd. This is particularly true in farm settings where purchased animals need to be screened or where segregation and culling of infected animals is much needed. Therefore, rapid and accurate pen-side diagnostic tests or combination of tests are needed. Given the difficulty in diagnosis due to the fastidious growth nature and subclinical infection, the disease can remain undetected for a long time in dairy farms with significant economic losses. Accurate diagnosis and antimicrobial susceptibility tests are also important to conduct targeted treatment which reduces the emergence of antimicrobial resistance. Whenever microbial culture is used for diagnosis, suspected animals awaiting results should be segregated from other herd members. Immediate culturing of milk samples is highly encouraged to increase the likelihood of detection since refrigeration and freezing lowers the survival of *Mycoplasma* cells (145). Cows with mastitis are usually asymptomatic and sometimes *M. bovis* is detected from healthy animals (146). This is also best exemplified by sudden occurrence of infection signs such as lameness and mastitis in yet closed dairy herds (147). Therefore, multiple sampling and regular screening of existing herd members is extremely important depending on how often new heifers are purchased. To overcome the problem of intermittent shedding of the *Mycoplasma* in the milk, indirect ELISA could be of great help since it depends on the antibodies rather than the detection of the antigen itself. MALDI-TOF is an advanced, rapid, and accurate method to detect *M. bovis* from various clinical samples. Its expensiveness and requiring sophisticated instrumentation and expertise renders it difficult to recommend for large-scale use. Finally, uniform recommendation should be put forward regarding which tests can be bundled together and yield complete diagnosis of *M. bovis* for different clinical samples collected from the animal body organs affected.

One of the major reasons for the failure to develop an effective control tool is limited knowledge of the virulence factors of *M. bovis* and the pathogenesis of *M. bovis* mastitis in dairy cows. Therefore, developing effective and sustainable control tools such as vaccines or prophylactic or therapeutic drugs, or any other innovative intervention tool using advanced molecular biology and cellular and molecular immunology approaches is required.

The combined economic and welfare impact of *M. bovis* infections prompted extensive search for effective and sustainable control tools such as vaccines, prophylactic and therapeutic solutions (21) while also antimicrobial resistance is increasing (20). Vaccine attempts has been futile due to mainly the knowledge gap in the pathogenesis and virulence mechanism of *M. bovis.* Novel vaccine or antimicrobial drugs will be out of reach if conserved immunogenic antigens or therapeutic agents are not discovered.

Hygienic husbandry practices during milking, feeding, and overall rearing is of paramount significance.

## Author contributions

AG: Writing – review & editing, Writing – original draft. SD: Writing – review & editing, Writing – original draft. BG: Writing – review & editing. OK: Writing – review & editing, Conceptualization, Supervision.
